# Placebo-controlled three-armed pilot trial of Escitalopram or Nortriptyline for depressive symptoms in Parkinson’s disease (ADepT-PD)

**DOI:** 10.1007/s00702-026-03170-8

**Published:** 2026-05-08

**Authors:** Anette Schrag, Andrew Embleton-Thirsk, Camille Carroll, Marc Serfaty, Gordon Duncan, Sophie Molloy, John Whipps, Blair McLennan, Glyn Lewis

**Affiliations:** 1 Department of Clinical and Movement Neurosciences, London, UK; 2 Comprehensive Clinical Trials Unit, University College London, London, UK; 3 Translational and Clinical Research Institute, National Institute for Health and Care Research (NIHR) Newcastle Biomedical Research Centre (BRC), Newcastle, UK; 4 Division of Psychiatry, UCL, London, UK; 5 NHS Lothian, Edinburgh, UK; 6 Department of Neurosciences, Imperial College Healthcare NHS Trust, London, UK; 7 PPI Representative, Plymouth, UK

**Keywords:** Parkinson’s Disease, Depression, Randomised Controlled Trial, Escitalopram, Nortriptyline, Placebo

## Abstract

**Abstract:**

Pilot randomised-controlled trial of nortriptyline and escitalopram compared to placebo for depressive symptoms in people with Parkinson’s disease (PD). Participants with PD and depressive symptoms received either nortriptyline, escitalopram or placebo with assessments at baseline and 8 weeks. Feasibility of the full trial was assessed using recruitment rate, loss to follow up before the 8-week primary endpoint, adherence to trial medication and rate of clinically significant adverse reactions. Intended efficacy outcomes included the BDI-II, Patient Health Questionnaire (PHQ-9), Parkinson Anxiety Scale (PAS), MDS-UPDRS and adverse effects. The aim was to recruit 46 participants to determine feasibility of the full trial. 52 participants were recruited from 24 NHS sites and randomised to nortriptyline (*n* = 16), escitalopram (*n* = 17) or placebo (*n* = 19). However, despite multiple strategies, recruitment took two years, and the study therefore did not reach its feasibility aim. Exploratory analyses showed that at 8 weeks depressive symptoms decreased significantly in all three arms. There was no difference in BDI-II score changes between the nortriptyline or the escitalopram when compared to the placebo arm, but PHQ-9 scores showed a greater reduction compared to placebo with nortriptyline (-3.4, 95% CI -5.75 to -0.95) but not with escitalopram (-1.2, 95% CI -3.54 to -1.17). Persistent Anxiety Scale scores showed a similar pattern (nortriptyline: -2.8, 95% CI -5.5 to -0.01; escitalopram: -2.2, 95% CI -4.9 to 0.05). As the study did not reach its primary aim of feasibility due to recruitment difficulties it did not continue to the full trial. The exploratory results are in keeping with previous studies suggesting a beneficial effect of nortriptyline on symptoms of depression and persistent anxiety. The small sample size however limits conclusions on efficacy and a beneficial effect of escitalopram is not excluded.

**Study dataset is available on ClinicalTrials.gov ID:**

NCT03652870, registered 29 August 2018

## Introduction

One of the most common complications of Parkinson’s disease (PD) are depressive disorders, which affect at least 30% of patients with PD (Reijnders et al. 2008) and are linked to functional impairment, cognitive decline and faster disease progression. Psychological therapies can be effective, but often antidepressant medications are required. Despite the high incidence of depression in this population, no conclusive evidence on appropriate choice of antidepressants in PD exists, and the risks of worsening of parkinsonism, adverse events, and aggravation of non-motor features of PD by antidepressants pose particular challenges in this population (Pina Latorre et al. 2001).

The most commonly used medications for the treatment of depressive disorders in the UK are selective serotonin re-uptake inhibitors (SSRIs). Tricyclic antidepressants (TCA), which have mixed properties including serotonin reuptake inhibition and noradrenaline reuptake inhibition as well as anticholinergic and antihistamine actions, have similar efficacy to SSRIs. The TCA nortriptyline has also been suggested to have neuroprotective properties in pre-clinical models of parkinsonian models (Collier et al. 2017). However, TCAs are currently only recommended as second line treatments for depression in PD due to their increased risk of adverse reactions including orthostatic hypotension, dry mouth, constipation, urinary retention, memory impairment, hallucinations and confusion.

Nevertheless, in depression in PD (dPD), TCAs have conventionally been used because their anticholinergic properties are considered beneficial for parkinsonian features, such as tremor, in early PD, and insomnia, with some trial evidence also supporting their efficacy for depressive symptoms in PD. SSRIs on the other hand, whilst also supported by some trial evidence, are sometimes used cautiously in dPD, as cases of new onset parkinsonism or worsening of parkinsonism have been reported (Pina Latorre et al. 2001). The reported effect of SSRIs on PD motor symptoms has varied between different studies, with some animal models and case reports suggesting parkinsonism as a reversible adverse effect of SSRIs, and suggesting deterioration of parkinsonism in some studies but not others. Additionally, other side effects such as fatigue, postural hypotension or falls can occur and may already be pre-existing in PD (Carriere et al. 2016), and very rarely serotonin syndrome has been reported. Therefore, there remains concern that SSRIs worsen Parkinson’s symptoms and that antidepressant treatment in dPD is not always clinically effective (Weintraub et al. 2003).

Although several systematic evidence reviews recommend the use of TCAs in dPD over SSRIs, there is a lack of large placebo-controlled comparative trials between these medication classes.

## Materials and methods

### Objective and study design

We conducted a internal pilot feasibility study for a three-arm, double-blind randomised trial in the UK with the overall aim to compare the effectiveness of the SSRI escitalopram and of the tricyclic nortriptyline to placebo to independently estimate the effects of each of the two medicines on depressive symptoms, anxiety and motor function as well as their safety in patients with PD (Schrag et al. 2022). The primary aim of the pilot study was to determine the feasibility of recruiting to a full trial, with an exploratory analysis being the comparison of outcomes in the nortriptyline vs. placebo and the escitalopram vs. placebo arms.

### Participants and recruitment

Participants were recruited from NHS Parkinson’s centres in the UK, and were randomly allocated through a web-based randomisation service to receive escitalopram or nortriptyline or placebo. Inclusion criteria were diagnosis of idiopathic PD, based on a history and neurological exam performed by the enrolling investigator with presence of at least two of the three cardinal signs of PD: rigidity, bradykinesia, and rest tremor with no evidence of diagnostic alternatives; age ≥ 18 years; fulfilling diagnostic (*DSM-V*) criteria for a depressive disorder (i.e. major depressive disorder or persistent depressive disorder) or operationally defined subsyndromal depression (presence of two or more depressive symptoms at threshold or subthreshold levels, at least one of which had to include depressed mood or anhedonia); Beck Depression Inventory II (BDI-II) (Beck et al. 1961) score ≥ 14; treatment with antiparkinsonian medication optimised or stable for at least 4 weeks before date of randomisation and with no plans to change in the primary assessment window (baseline to 8 weeks). Exclusion criteria were Montreal Cognitive Assessment (MoCA) (Nasreddine et al. 2005) score < 16 or lack of capacity to consent; women who were pregnant, breastfeeding or of childbearing potential without effective contraception; insufficient understanding of the English language or not able to understand the Participant Information Sheet or the self-completed questionnaires; treatment with an antidepressant within 4 weeks of enrolment (except for a small dose of amitriptyline up to 30 mg for indications other than depression); known severe liver failure; absolute contraindications to escitalopram or nortriptyline (known QT-interval prolongation or congenital long QT syndrome, recent myocardial infarction (< 3 months), any degree of heart block or other cardiac arrhythmias precluding treatment with nortriptyline or escitalopram according to clinical judgement); medications contraindicated on nortriptyline or escitalopram; active suicidal ideation or intent on the BDI-II item 9 and who, after clinical review of risk using the standardised Suicide Risk Management Protocol, need to be referred for immediate treatment; participation in another clinical trial of an investigational medicinal product or device within 30 days of randomisation; any clinical condition which in the opinion/clinical judgement of the investigator would make the patient unsuitable for the trial due to safety concerns.

The study was undertaken in compliance with the Declaration of Helsinki and fully informed consent was obtained from all participants. Ethical approval was granted by the London – Riverside Research Ethics Committee (REC) number 19/LO/0288. The study was registered on ClinicalTrials.gov with Identifier NCT03652870. Enrolment began 22 March 2021 and ended 30 September 2022.

### Intervention

Following consent, participants were randomised, stratified by centre and severity of depression to oral escitalopram (target dose 20 mg in patients aged ≤ 65, or 10 mg in patients aged > 65 or those with hepatic impairment) or nortriptyline (target dose 100 mg in patients aged ≤ 65, or 50 mg in patients aged > 65 or those with hepatic impairment) or placebo. The initial dosage of trial medication was either 5 mg escitalopram increased by 5 mg per day, at two weekly intervals, to a maximum target of 20 mg escitalopram per day, or 25 mg nortriptyline increased by 25 mg per day, at two-weekly intervals, to a maximum target of 100 mg nortriptyline per day or placebo. If intolerable side effects occurred, the dosage could be decreased to the previous dose level. To maintain study blinding, all trial medication was provided in bottles of identically appearing tablets. Site staff completing assessments were kept masked to trial arm allocation, as were the participants and the trial team. Participants were asked to record uptake and timing of trial medication using a dosing diary, with reminder phone calls at escalation points, and their dose level was recorded at the 8-week timepoint.

### Primary outcome

Assessment of feasibility of the full trial was based on recruitment rate, loss to follow up before the 8-week primary endpoint, adherence to trial medication and rate of clinically significant adverse reactions. The target enrolment for progression from the pilot phase was 46 participants recruited in 6 months.

### Exploratory outcomes

The planned primary outcome for the full trial was change in depressive symptoms after 8 weeks of treatment as measured using the BDI-II against placebo; this was an exploratory outcome in the pilot phase. Other outcomes included the Patient Health Questionnaire 9 (PHQ-9) (Kroenke et al. 2001), the Parkinson Anxiety Scale (PAS) (Leentjens et al. 2014), the Movement Disorder Society Unified Parkinson’s Disease Rating Scale (MDS-UPDRsS) part I-IV (Goetz et al. 2008), the EQ-5D-5 L (Herdman et al. 2011), Clinical Global Impression (CGI) and the Modified Toronto Side Effect Scale (Crawford et al. 2014) score plus reporting of adverse events. Any event that met the criteria for an adverse event (AE) and serious adverse event (SAE) was recorded.

### Statistical analysis of the exploratory outcomes

Descriptive analyses were used to examine the baseline characteristics of the treatment groups by randomised group. All applicable statistical tests were 2-sided. All analyses were performed in Stata version 18. Following database-lock, the outcome analysis was conducted following the intention to treat (ITT) principle according to a previously agreed Statistical Analysis Plan. A generalised linear mixed effects model was used to estimate the outcome effect of the difference in BDI-II score, plus the other exploratory outcomes, at 8 weeks post baseline between the treatment groups. The mixed-effects model, separate for the two active treatment-to-placebo comparisons, included fixed effects for treatment, time (incorporating the baseline and follow up measurements), the treatment-time interaction, and random effects for individual participant identifiers and site.

### Safety analysis

We describe the number of participants experiencing adverse events on the Modified Toronto Side Effects Scale, which assesses potential side effects in patients taking antidepressant medications, and the number of adverse events plus drop-outs.

### Sample size calculation

The purpose of the pilot trial was to determine the feasibility of a definitive study to assess efficacy. Power calculations for the intended full trial indicated that for 90% power and a significance level of 0.025 (for each comparison to preserve studywise alpha), 113 participants would be needed per group to detect a 3-point BDI-II difference [SD for change 6.35] for the escitalopram–placebo and the nortriptyline–placebo comparisons at 8 weeks. Allowing for 20% attrition, an overall sample size of 408 participants would be required. Predefined progression criteria for the trial were based on recruitment rate, adverse events, loss to follow-up before primary endpoint and adherence to trial medication.

**Fig. 1:**
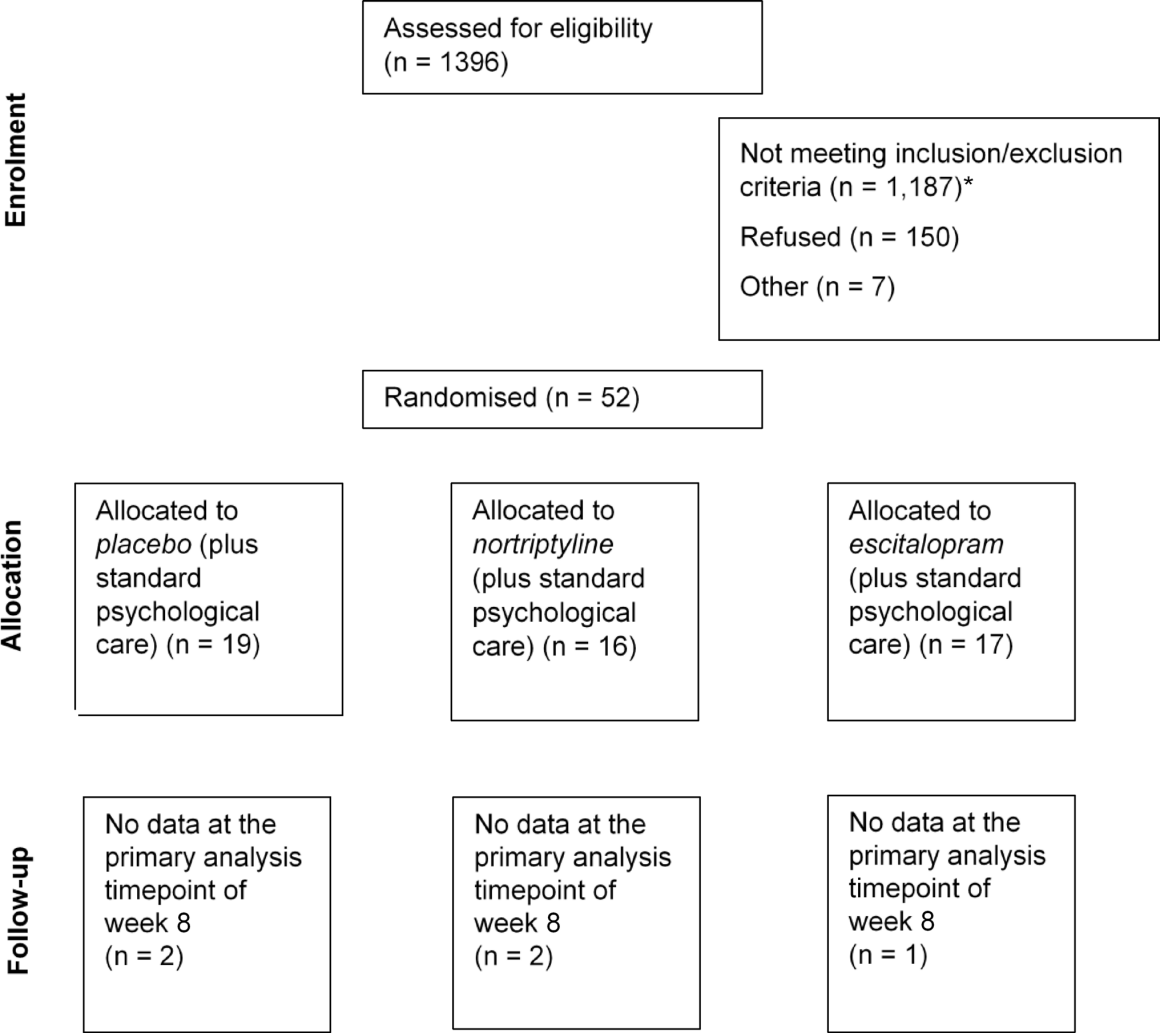
Consort Flow Diagram.*at pre-screening, patients without documented depressive features were not excluded; the main reasons for screen failure were not fulfilling inclusion criteria for depressive features at the in-person visit, treatment with an antidepressant within 4 weeks of enrolment, refusal to participate and not fulfilling criteria for PD, followed by antiparkinsonian treatment not stable, having absolute contraindications to one of the trial medications, not in the inclusion age range, and low MoCA score.

**Fig. 2:**
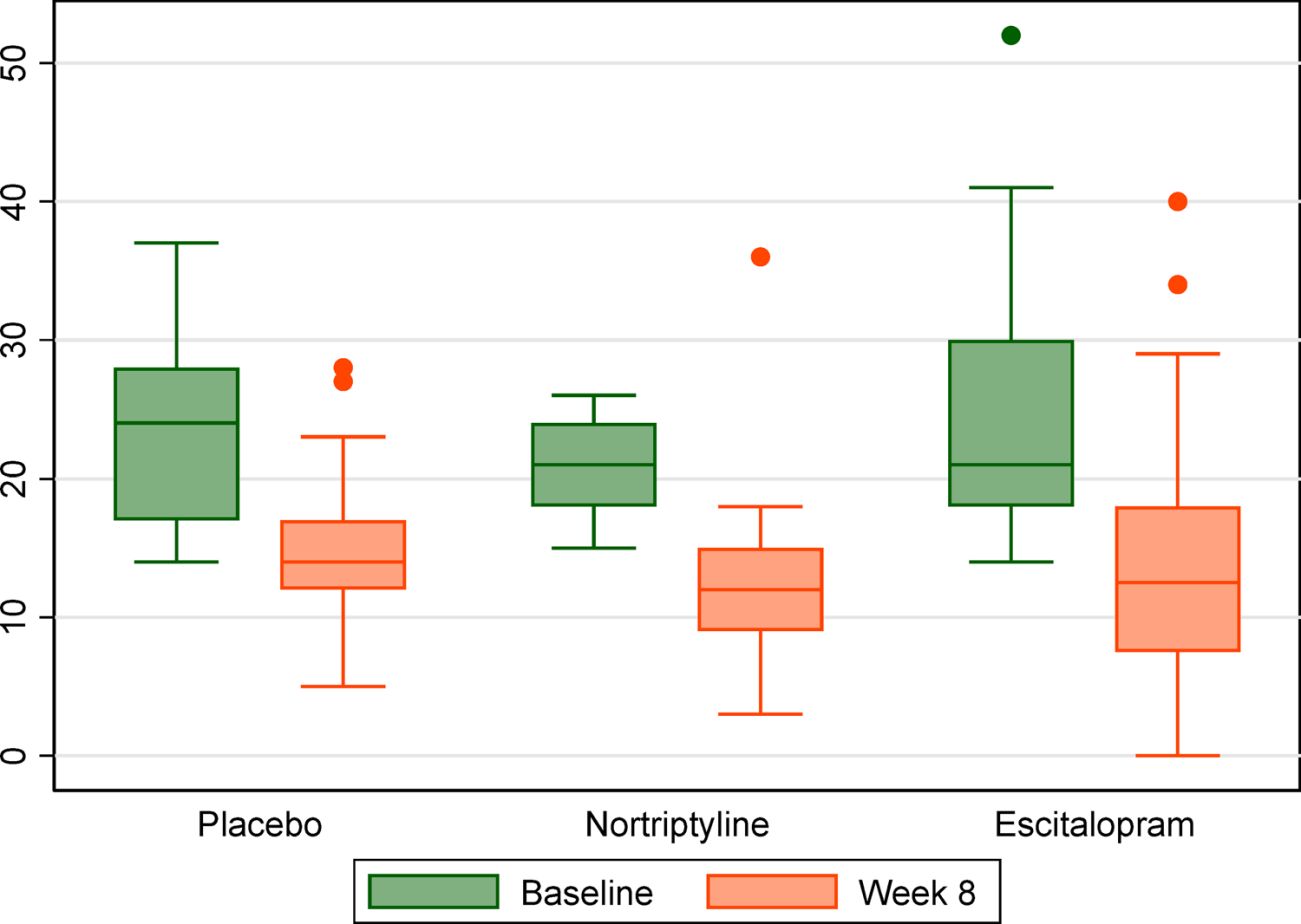
BDI-II total score at baseline and Week 8, by allocated arm

## Results

Overall, 52 participants from 24 NHS sites were randomised, 16 to the nortriptyline arm, 17 to the escitalopram arm and 19 to the placebo arm (Fig. 1; Table 1). There was a high number of screen failures (Fig. 1). The main reason was not fulfilling inclusion criteria for depressive features at the in-person visit, as we purposefully did not exclude participants without a previous record of depressive features before screening. Other reasons for screen failures included antidepressant use within 4 weeks of enrolment, refusal to participate, and not fulfilling criteria for PD, followed less commonly by antiparkinsonian treatment not stable, having absolute contraindications to one of the trial medications, not being in the inclusion age range, and low MoCA score. Study participants had a mean age of 62 (SD 10.7), 16 were female (30.8%) and 36 (69.2%) were male. The number of patients > 65 years was 25 (48%); no participant had hepatic impairment. At baseline, 19 participants had BDI-II scores between 14 and 19, indicating mild depressive symptoms and 33 participants had BDI-II scores of 20–63, indicating moderate to severe depressive symptoms (Supplementary Table 1).

**Table 1:** Participant characteristics at study entry

	Placebo (*N* = 19)	Nortriptyline (*N* = 16)	Escitalopram (*N* = 17)	Total (*N* = 52)
Gender:				
Female; N (%)	5 (26.3)	5 (31.3)	6 (35.3)	16 (30.8)
Male; N (%)	14 (73.7)	11 (68.8)	11 (64.7)	36 (69.2)
Age [years]; mean (sd)	63 (11.7)	63 (11.3)	61 (9.4)	62 (10.7)
Age category				
Under 65; N (%)	8 (42%)	8 (50%)	11 (65%)	27 (52%)
65 and over; N (%)	11 (58%)	8 (50%)	6 (35%)	25 (48%)

### Primary outcome

#### Feasibility to recruit

The study faced considerable recruitment difficulties, with a high number of screen failures and interruption by the COVID-19 pandemic. Early on, due to the pandemic. we changed the trial protocol to make the trial deliverable completely remotely. Considerable efforts were made to increase recruitment rates, including the opening of multiple identification and recruitment sites across the UK, provision of materials for sites to explain the purpose and methods of the trial in accessible format, publicity through the major Parkinson’s charities in the UK, the Clinical Research Network and social media, recruitment from primary, secondary and tertiary care centres as well as Mental Health Trusts, and amendments to the protocol to increase the eligible age range. Nevertheless, recruitment in the pilot phase took 24 months, much slower than the target of 46 within an originally planned 6-month pilot phase, even accounting for the COVID-19 pandemic. The primary outcome of feasibility of a full trial was therefore not reached, and the study was closed after completion of this phase.

#### Adverse events

Adverse Events were mostly mild (Supplementary Table 9). There was no difference between arms in adverse event number (*p* = 0.75) or grade experienced (*p* = 0.37; Supplementary Tables 9 - Supplementary Table 13). Two Serious Adverse Events (SAEs) occurred on trial, both being in the same participant in the escitalopram arm (urinary tract infection and spinal infection, both judged to be unrelated to the treatment). There was no difference in the scores on the Modified Toronto Side Effects (Supplementary Table 7) in either the nortriptyline versus placebo comparison (*p* = 0.96) nor the escitalopram comparison (*p* > 0.99) in the total score at 8 weeks.

#### Loss to follow-up before primary endpoint

Four participants withdrew prior to the primary endpoint, two in the placebo arm and two in the nortriptyline arm, one of whom withdrew prior to administration of the trial medication. One other participant in the escitalopram arm missed the primary endpoint assessment (Fig. 1).

#### Adherence to trial medication

At 8 weeks, only 25 participants had reached the maximum level of the trial medication (8 each in the nortriptyline and in the escitalopram arm and 9 in the placebo arm).

### Exploratory outcomes

#### Depression and anxiety outcomes

All three groups had decreases in their BDI-II severity between baseline and eight-weeks (Supplementary Table 2, Fig. 2). There were no statistically significant differences between the groups: the mean difference for change in BDI-II severity was − 3.1 (95% confidence interval (CI) -8.66 to 2.53, *p* = 0.28) for nortriptyline vs. placebo and − 0.7 (95% CI -6.11 to 4.70, *p* = 0.80) for escitalopram vs. placebo (Supplementary Table 3). Five participants had no data at 8 weeks, two each in the placebo and nortriptyline arms, and one in the escitalopram. These participants were excluded from the model. A sensitivity analysis including their baseline scores had only very limited effect on the estimates. There was a decrease in depression scores in the PHQ-9 outcome measure in all arms (Table 2) with a mean change from baseline to eight weeks of -2.6 (SD 4.67) in the nortriptyline arm, a change of -4.6 (SD 5.00) in the escitalopram arm and − 2.7 (SD 3.93) in the placebo arm (Supplementary Table 4). The mean difference in PHQ-9 change over 8 weeks compared to placebo was − 3.4 (95% CI -5.75 to -0.95, *p* = 0.01 in the nortriptyline arm, and − 1.2 (95% CI -3.54 to 1.17, *p* = 0.33) in the escitalopram arm vs. placebo.

**Table 2:** Patient Health Questionnaire (PHQ-9) score

Total; mean (sd) [*n*]	Placebo (*N* = 19)	Nortriptyline(*N* = 16)	*p*-value Nortriptyline vs. Placebo	Escitalopram (*N* = 17)	*p*-value Escitalopram vs. Placebo
Baseline	12.7 (3.4)	9.1 (2.6)	0.01	11.9 (5.2)	0.33
8 week	9.6 (3.7)	6.7 (3.5)		7.3 (5.5)	
Change (in complete cases)	–2.7 (3.93)	-2.6 (4.67)		-4.6 (5.00)	

Please note, baseline values are presented for all 52 participants, whilst the difference is calculated for the 47 participants with 8 week data

There was also a significant decrease in the Persistent PAS subscale in the nortriptyline arm (*p* = 0.03) but not the escitalopram arm (*p* = 0.10) compared to placebo at 8 weeks but the decrease in the total Parkinson’s Anxiety Scale was not statistically significant in either the nortriptyline (*p* = 0.06) or the escitalopram arm (*p* = 0.15), nor the Episodic or Avoidance Subscales (Table 3 and Supplementary Table 5).

**Table 3:** Parkinson Anxiety Scale and Subscales

	Total; mean (sd) [n]	Placebo (*N* = 19)	Nortriptyline (*N* = 16)	p-value Nortriptyline vs. Placebo	Escitalopram (*N* = 17)	p-value Escitalopram vs. Placebo
Persistent	Baseline	11.5 (4.2)	9.2 (3.8) [16]	0.03	9.6 (5.2)	0.10
	8 week	8.5 (3.9)	5.1 (4.4) [14]		6.3 (4.5)	
Episodic	Baseline	4.4 (3.3)	2.7 (2.9) [16]	0.17	3.5 (3.2)	0.46
	8 week	2.2 (2.3)	1.6 (2.5) [14]		1.8 (2.8)	
Avoidance	Screening	4.4 (3.3)	2.7 (2.9) [16]	0.55	3.5 (3.2)	0.41
	8 week	2.2 (2.3)	1.6 (2.5) [14]		1.8 (2.8)	

There was no significant difference of MDS-UPDRS part III scores between the nortriptyline or the escitalopram arm and the placebo arm although there was a trend towards a greater reduction of scores in the nortriptyline arm (*p* = 0.09). There was no difference in change of MDS-UPDRS part I, II or IV between treatment arms (Supplementary Table 6), of the MoCA for either nortriptyline (*p* = 0.23) or escitalopram (*p* = 0.78; Supplementary Table 7), the CGI and the EQ-5D-5L utility and VAS scores.

## Discussion

This three-arm internal pilot randomised controlled trial of depression in patients with Parkinson’s disease was unable to recruit sufficient participants in a timely manner, making it clear that recruitment to a full trial is not feasible. The overall study sample size for an effectiveness trial was planned to be 408 participants, but it became clear during the pilot study that this could not be achieved as recruitment of this population was difficult. This is likely due to a number of reasons, suggested by the reasons for screen failures, including the high proportion of patients with depression in PD that were already treated with antidepressants and therefore not eligible, patients not willing or unable to take antidepressants, reluctance or inability to participate in a trial when already burdened with complex disease symptoms and a medication regime that requires optimisation, and the high number of potential contraindications for either of the study medications. Further complicating factors were the COVID19 pandemic resulting in low recruitment and research staff shortages, and the availability of one of the trialled antidepressants (due to interruption of supply by the manufacturer). Despite putting in place multiple strategies to enhance recruitment, including allowing for completely remote recruitment and assessment with direct-to-participant postal delivery of the trial medication, publicising the study by the main Parkinson’s disease charities in the UK and through the Clinical Research Network, training and recommendations to identify depression in people with PD during and before clinical appointments, and increasing the eligibility window for age, recruitment was not sufficient to allow for continuation to the planned full trial. Nevertheless, in participants recruited into the study there was no indication of any significant safety issues, suggesting that in PD for patients who are depressed and who do not have contraindications, these medications are tolerated. Neither the tricyclic nortriptyline nor the SSRI escitalopram was associated with safety concerns in this pilot study, although exclusion of a number of patients based on existing comorbidities excluded a number of participants at screening.

The study was not powered to assess efficacy of either nortriptyline or escitalopram, and the sample size was too small to evaluate the overall effectiveness of these medications. Furthermore, whilst retention was reasonable at 8 weeks, only 25 participants reached the target dose. This short period was chosen in order to allow for an assessment of efficacy, and for the opportunity to switch to another treatment if no effect is seen at this time point, in line with clinical practice. However, this time frame may be too short to reach effective doses in this population and an increase in the titration period may have allowed for higher dose titration. Nevertheless, the exploratory findings were in keeping with their expected effects on depressive and anxiety symptoms. The BDI-II scores decreased in all three arms, but there was no difference between either medication compared to placebo with wide confidence intervals for the differences in BDI-II total scores. These confidence limits included values that would be consistent with a clinically important treatment effect for both medications. We did find some evidence of a difference in depressive symptoms using the PHQ9 between the nortriptyline and the placebo arm at 8 weeks, suggesting that this might be a more sensitive measure to detect changes in depression scores in patients with PD than the BDI-II, though caution is advised as no adjustment for multiplicity was utilised. This result is in keeping with the relative responsiveness of these instruments in people with depression without PD (Kounali et al. 2016). Furthermore, there was an improvement in Persistent Anxiety measured on the Parkinson’s Anxiety Scale in the nortriptyline arm compared to placebo, with no evidence for a difference in the escitalopram arm. Despite the limitations in sample size and some differences in baseline values between groups, these results are broadly in keeping with previous reports of a subtle response in depression scores to tricyclics at 8 weeks in patients with PD. Menza et al. reported a significant improvement depression scores on the Hamilton Depression Inventory in 17 patients on nortriptyline with no significant improvement in 18 patients in the paroxetine arm (Menza et al. 2009). Devos et al., on the other hand found that both desipramine and citalopram had improved depression scores after 30 days (Devos et al. 2008), and Richard et al. reported in a larger trial of 112 participants that both venlafaxine and paroxetine improved depression scores (Richard et al. 2012). The severity of depression in these studies may also have been higher although comparisons are difficult to make due to the use of different outcome measures for depression.

The results provide some preliminary evidence of the tolerability of these medications in patients with PD and potential effectiveness of nortriptyline for depressive and persistent anxiety symptoms in PD. Given the challenges in recruitment, future trials of depressive medications in PD should limit randomisation to two arms to reduce the required sample size and to reduce the number of contraindications. Trials for this indication may also be more attractive for potential participants and their clinical teams if they trial antidepressant medications that may offer additional benefits, have fewer contraindications or examine non-pharmacological options. Trials including only participants with more severe depressive features would reduce required sample sizes but recruitment and retention may be more challenging. Furthermore, trials with the studied or related medications for different indications, e.g. potential neuroprotective effects, which may include a less selected population of people with PD, i.e. not only those with depressive features, are likely to be able to recruit higher number of participants.

## Data Availability

The data that support the findings of this study are available on request from the corresponding author. The data are not publicly available due to privacy or ethical restrictions.
